# The Role of Olive Oil Polyphenols in Osteosarcopenic Obesity-Related Biological Domains: A Systematic Review of Current Evidence

**DOI:** 10.3390/foods14162766

**Published:** 2025-08-08

**Authors:** Roberta Zupo, Fabio Castellana, Maria Lisa Clodoveo, Giuseppe Lisco, Giuseppe Mazzola, Mariangela Rondanelli, Alice Cantù, Patrizia Riso, Simone Perna

**Affiliations:** 1 Department of Interdisciplinary Medicine (DIM), University of Bari Aldo Moro, Piazza Giulio Cesare 11, 70100 Bari, Italy; castellanafabio@gmail.com (F.C.); marialisa.clodoveo@uniba.it (M.L.C.); giuseppe.lisco@uniba.it (G.L.); 2 Endocrinology and Nutrition Unit, Azienda di Servizi alla Persona “Istituto Santa Margherita”, University of Pavia, 27100 Pavia, Italy; giuseppe.mazzola02@universitadipavia.it; 3 Department of Public Health, Experimental and Forensic Medicine, University of Pavia, 27100 Pavia, Italy; mariangela.rondanelli@unipv.it; 4 Department of Food, Environmental and Nutritional Sciences, University of Milan, 20133 Milan, Italy; alice.cantu1@studenti.unimi.it (A.C.); patrizia.riso@unimi.it (P.R.); simone.perna@unimi.it (S.P.)

**Keywords:** osteosarcopenic obesity, olive oil, polyphenols, hydroxytyrosol, oleuropein, adipogenesis, osteoporosis, sarcopenia

## Abstract

Background: Osteosarcopenic obesity (OSO) is an emerging syndrome characterized by the coexistence of obesity, sarcopenia, and osteoporosis, primarily affecting aging populations. Nutrition, especially polyphenol-rich foods like extra virgin olive oil (EVOO), may play a preventive or therapeutic role in OSO. This review aims to critically examine evidence from in vitro, in vivo, and human studies on the effects of olive oil polyphenols on OSO-related biological domains. Methods: This systematic review followed PRISMA guidelines. Studies were identified from PubMed and Google Scholar using MeSH terms related to olive oil, polyphenols, and OSO-associated conditions. In vitro and in vivo studies (both in animal and human models) published in the last ten years were included. The study protocol was registered with PROSPERO (CRD420251077836). Results: Fifteen studies were included: eight in vitro, four in vivo on animal models, and three human trials. Phenolic compounds such as hydroxytyrosol, oleuropein, oleocanthal, and oleacein demonstrated antioxidant, anti-inflammatory, anti-adipogenic, and osteo-/myo-protective effects. These compounds modulated key metabolic pathways and gene expression related to adipogenesis, bone metabolism, and muscle integrity. Conclusions: Olive oil polyphenols exhibit promising biological effects on the tissues involved in OSO. Although evidence is mostly preclinical, selected compounds (notably hydroxytyrosol and oleuropein) may serve as adjuncts in nutritional strategies for OSO prevention.

## 1. Introduction

Despite increasing recognition of the individual benefits of olive oil polyphenols on bone, muscle, and adipose tissue, their comprehensive role in addressing osteosarcopenic obesity (OSO)—a syndrome involving simultaneous degeneration of all three tissues—remains underexplored. Most existing studies assess polyphenol effects in isolation, without considering the complex interrelations that define OSO. Furthermore, no previous review has systematically synthesized evidence across preclinical and clinical models to evaluate the potential of these compounds as multi-target interventions. This knowledge gap hampers the development of nutritionally based strategies to mitigate OSO in aging populations.

As the global population ages, promoting health and physical activity while reducing frailty and nutritional deficiencies becomes a critical challenge. Aging induces major physiological changes, especially in body composition. Sarcopenia, the loss of skeletal muscle mass and strength, begins in midlife and worsens with age, raising fall and frailty risks [1]. Bone mass also peaks at approximately 30 years old, declining with age, especially in postmenopausal women due to reduced estrogen levels, increasing fracture risk. Conversely, fat accumulation rises with age, redistributing toward visceral and ectopic depots, impairing musculoskeletal function, and promoting inflammation [2]. The co-occurrence of osteoporosis, sarcopenia, and obesity defines osteosarcopenic obesity (OSO) [3], a systemic syndrome linked to aging, inactivity, inflammation, and poor diet. Endocrine and chronic diseases may further aggravate it. Diagnosis relies on body composition and functional assessment [4]. A systematic review of 20 studies (*n* = 23,909) estimated OSO prevalence at 8%, with higher rates among women and older adults [5]. Regional studies confirm this trend: in the United States, prevalence in older community-dwelling adults reaches up to 11.6% [6]; in Korea and China, estimates range from 5% to 13%, depending on sex and diagnostic criteria [7]. Italian studies report 1.9% prevalence in the general population and up to 6.79% among bedridden elderly [8]

Prevention strategies for OSO primarily rely on preserving bone and muscle mass while limiting fat accumulation through established interventions such as resistance and aerobic exercise, adequate protein, calcium, vitamin D, and polyunsaturated fatty acids (PUFAs) [8]. In contrast, the potential contribution of food-derived bioactive compounds—such as polyphenols—remains speculative and less investigated. These compounds may exert pleiotropic effects on adipose, bone, and muscle tissue, but their role in the integrated context of OSO has not yet been fully elucidated [9]. *Olea europaea L.*, native to the Mediterranean, is cultivated for olive oil via mechanical extraction. According to EU/IOC standards, EVOO is the highest-grade oil (acidity < 0.8%) and is unrefined [9]. It contains mainly triacylglycerols and MUFA (≈85%, primarily oleic acid), linoleic, palmitoleic, and saturated fats. Minor bioactives (≈2%) include phenolic acids, flavonoids, secoiridoids, tocopherols, carotenoids, and pectins [10]. Their concentration depends on processing and storage. EVOO polyphenols include secoiridoids (oleacein, oleuropein), phenylethanols (hydroxytyrosol, tyrosol, oleocanthal), and phenolic acids [11,12,13,14]. Hydroxytyrosol (HT) is a potent antioxidant and anti-inflammatory compound, with additional antimicrobial and anticancer effects [15]. Oleocanthal and oleacein have anti-inflammatory, antiplatelet, and neuroprotective effects [16]. EVOO polyphenols are stable in gastric conditions and rapidly absorbed: HT peaks in plasma within 5–10 min, oleuropein in ≈2 h [17]. Extraction methods influence their concentration; de-pitted olive oil retains more oleocanthal. These polyphenols show multifunctional activities relevant to OSO and may represent nutritional tools for its prevention.

While extra virgin olive oil (EVOO) remains the most commonly consumed source of olive polyphenols, the current literature also includes evidence from other *Olea europaea L.*-derived sources such as olive leaf extracts, purified phenolic compounds, and complex standardized formulations. Therefore, this systematic review aims to examine the effects of polyphenolic compounds derived from *Olea europaea L.* (Intervention), compared to placebo or standard care (Comparator), on adipose tissue metabolism, bone health, and skeletal muscle integrity (Outcomes) in adult populations and experimental models relevant to osteosarcopenic obesity (Population).

## 2. Methods

This systematic review adhered to the Preferred Reporting Items for Systematic Reviews and Meta-Analyses (PRISMA) guidelines (PRISMA Guidelines). Eligibility criteria were defined a priori. Studies were included if they investigated the effects of polyphenolic compounds derived from *Olea europaea* L. (e.g., olive oil and olive leaf extracts) on biological domains related to osteosarcopenic obesity (OSO), including adipose tissue inflammation and metabolism, bone health, and skeletal muscle integrity. Studies were excluded if they used non-mammalian cell lines (e.g., plant, bacterial, or insect models) in in vitro experiments, or if they lacked original data. Additionally, non-peer-reviewed publications, such as conference abstracts, preprints, theses, and editorials, were not considered. This encompassed studies on OSO itself or its components (sarcopenia, osteopenia, osteoporosis, obesity) in adult participants (≥18 years) or relevant in vitro and in vivo models. Interventions involved isolated bioactives (e.g., hydroxytyrosol and oleuropein) or complex formulations, with comparative designs (placebo, standard care, or alternative interventions). Outcomes included quantitative changes in skeletal muscle mass or function, bone mineral density (BMD) or remodeling markers, fat mass and distribution, metabolic biomarkers (e.g., glucose, insulin, lipid profiles, and inflammatory cytokines like IL-6), or physical health indicators. Randomized controlled trials (RCTs), cohort and case–control studies, and mechanistic in vitro and in vivo studies were eligible. Studies lacking original data, not published in English, or not reporting OSO-related outcomes were excluded.

The following Boolean search string was used in Google Scholar: “olive oil” OR “Olea europaea” AND “polyphenols” AND (“osteosarcopenic obesity” OR “sarcopenia” OR “osteoporosis” OR “obesity”) AND (“muscle mass” OR “bone density” OR “fat mass”). Given the high volume of returns, only the first 300 results—ranked by relevance—were screened, as recommended in literature to balance feasibility and completeness when using non-specialized databases. Boolean operators refined the results. Reference lists of included studies and reviews were manually screened. Although only two databases (PubMed and Google Scholar) were used, this was a deliberate methodological choice to focus on peer-reviewed biomedical literature and maximize specificity for polyphenol-related interventions. To minimize selection bias, we performed an extensive manual screening of the reference lists of eligible articles and relevant reviews. Future reviews may benefit from incorporating additional structured databases such as Embase, Scopus, or Cochrane CENTRAL to ensure broader coverage. The study protocol was registered with PROSPERO (CRD420251077836).

## 3. Risk of Bias Assessment

**Figure 1:**
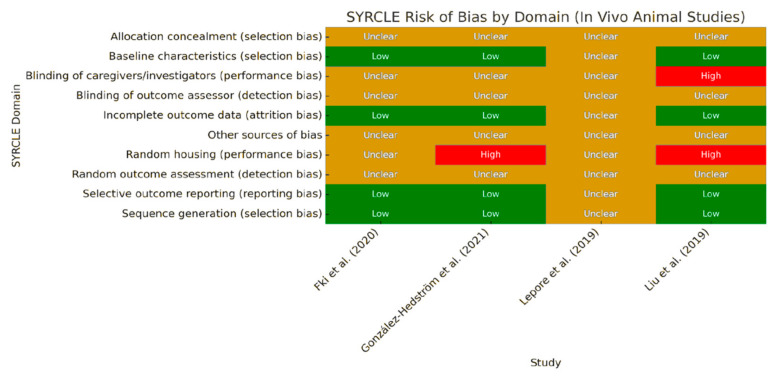
SYRCLE’s Risk of Bias assessment across in vivo animal studies. The heatmap summarizes the risk of bias across ten methodological domains. Green = low risk; red = high risk; yellow = unclear. Abbreviations: AC = Allocation Concealment (selection bias); Bl of C = Baseline Characteristics (selection bias); Bl of OA = Blinding of Outcome Assessor (detection bias); IOD = Incomplete Outcome Data (attrition bias); OSB = Other Sources of Bias; RH = Random Housing (performance bias); ROR = Random Outcome Assessment (detection bias); SOR = Selective Outcome Reporting (reporting bias); SG = Sequence Generation (selection bias). Data adapted from Lepore et al. [19], Liu et al. [20], Fki et al. [21], González-Hedström et al. [22].

For in vivo animal studies, risk of bias was assessed using SYRCLE’s Risk of Bias Tool (version 1.0), as described by Hooijmans et al. [18], which includes ten domains related to selection, performance, detection, attrition, and reporting bias. Two reviewers independently evaluated each domain and classified it as ‘low’, ‘high’, or ‘unclear’ risk based on the methodological details reported. The results were visualized through a heatmap summarizing the risk levels across studies and domains (Figure 1).

**Figure 2:**
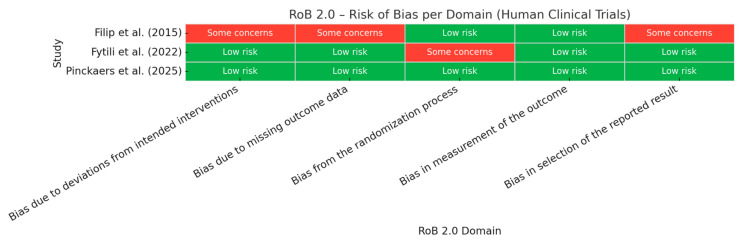
Heatmap comparing risk assessments across domains of human studies using the Cochrane Risk of Bias 2.0 (RoB 2.0). Data adapted from Fytili et al. [23], Pinckaers et al. [24], Filip et al. [25].

The risk of bias in randomized clinical trials was assessed using the Cochrane Risk of Bias 2.0 (RoB 2.0) tool, which evaluates five domains: (1) bias arising from the randomization process, (2) bias due to deviations from intended interventions, (3) bias due to missing outcome data, (4) bias in measurement of the outcome, and (5) bias in selection of the reported result. Two reviewers independently evaluated each domain and categorized as low risk, some concerns, or high risk. A summary heatmap was generated to visually compare risk assessments across domains and studies (Figure 2).

**Figure 3:**
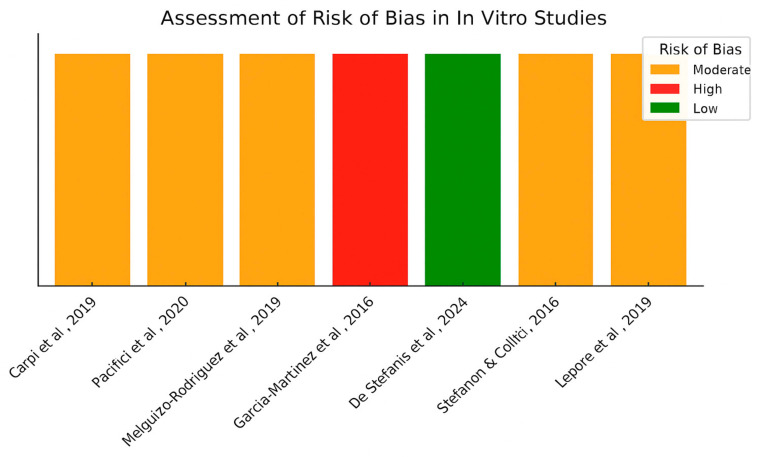
Risk assessment across selected in vitro studies using a structured grid adapted from the ToxRTool (Toxicological data Reliability Assessment Tool) and the OHAT Risk of Bias Tool developed by the U.S. National Toxicology Program. Data adapted from Stefanon and Colitti [27], Lepore et al. [19], Carpi et al. [28], Pacifici et al. [29], Melguizo-Rodríguez et al. [30], Garcia-Martínez et al. [31], De Stefanis et al. [32].

Finally, the risk of bias in the included in vitro studies was assessed using a structured grid adapted from the ToxRTool (Toxicological data Reliability Assessment Tool) [26] and the OHAT Risk of Bias Tool developed by the U.S. National Toxicology Program. Ten methodological domains were considered: (1) clarity of cell model selection, (2) presence of adequate experimental controls, (3) description and number of biological and technical replicates, (4) transparency and reproducibility of methods, (5) randomization of experimental layout, (6) blinding of outcome assessment, (7) appropriateness of statistical analyses, (8) use of validated protocols or standards, (9) transparency in reporting funding sources and conflicts of interest, and (10) completeness of outcome reporting. Each domain was scored qualitatively (Yes/No) based on explicit statements in the articles. Each study received a score corresponding to the number of domains rated as “Yes” (i.e., meeting methodological standards). Final classification was based on the total number of positive ratings: studies scoring 8–10 was considered low risk of bias, 5–7 as moderate risk, and 0–4 as high risk. No weighting scheme was applied (Figure 3).

## 4. Results

A total of 15 studies were included in this systematic review: 8 in vitro, 4 in vivo using rodent models, and 3 human clinical trials. Although the literature search was conducted up to 31 March 2024, one randomized controlled trial was included as it became available online ahead of print in April 2024 during the final data extraction phase, and met all predefined eligibility criteria. Table 1(A,B) summarize the current preclinical and clinical studies evaluating the effects of olive oil polyphenols on OSO-related outcomes. To enhance clarity and comparability, the studies were categorized not only by model type and key outcomes, but also according to the compound investigated. Specifically, Table 1(A) includes studies evaluating the effects of purified polyphenols (e.g., hydroxytyrosol, oleuropein, and oleocanthal), while Table 1(B) reports studies involving crude or complex mixtures such as extra virgin olive oil extracts and olive leaf extracts. Hydroxytyrosol was studied by Stefanon and Colitti [27] while Lepore et al. [19] focused on oleacein, and Carpi et al. [28] examined both oleacein and oleocanthal. Pacifici et al. [29] investigated tyrosol, and Melguizo-Rodríguez et al. [30], as well as Garcia-Martínez et al. [31], evaluated a range of phenolic compounds. De Stefanis et al. [32] studied oleocanthal and Nardi et al. [33] assessed oleuropein. In vivo studies included work by Liu et al. [20] on oleuropein, Liu et al. [34] on hydroxytyrosol, and Fki et al. [21] on both compounds. González-Hedström et al. [22] evaluated a complex mixture of phenolics. The three human studies included hydroxytyrosol [23], oleuropein [24], and a standardized polyphenol extract [25]. These compounds exert various biological activities, influencing pathways relevant to OSO.

### 4.1. Effects on Adipose Tissue

In vitro and in vivo studies consistently demonstrated the beneficial effects of polyphenols on adipose tissue. Carpi et al. [28] suggested that oleocanthal and oleacein may reduce adipocyte inflammation by downregulating IL-1β, COX-2, and miRNA expression via NF-κB inhibition, while enhancing PPARγ. Pacifici et al. [29] found that tyrosol suppresses adipogenesis and promotes lipolysis through the AMPK-ATGL-HSL pathway. Lepore et al. [19] reported that oleacein attenuated adipocyte hypertrophy and inflammation by reducing FAS and SREBP-1 while increasing adiponectin. Stefanon and Colitti [27] found that hydroxytyrosol was associated with decreased triglyceride accumulation and induces apoptosis in preadipocytes by modulating gene expression. In vivo, Fki et al. [21] observed reduced body weight and adiposity in rats administered hydroxytyrosol or oleuropein, particularly with hydroxytyrosol reducing leptin and TNF-α. Liu et al. [34] showed that hydroxytyrosol reshaped gut microbiota composition and suppressed inflammation via NF-κB inhibition. In human trials, Fytili et al. [23] demonstrated that hydroxytyrosol supplementation (15 mg/day) significantly reduced visceral fat and improved lipid metabolism.

### 4.2. Effects on Bone Tissue

Phenolic compounds also displayed osteoprotective effects. Melguizo-Rodríguez et al. [30] found that several EVOO phenolics upregulated osteogenic markers such as TGFβ1, BMP2, and BMP7. Garcia-Martínez et al. [31] confirmed that hydroxytyrosol and select phenolic acids enhanced osteoblast proliferation, with the most effective extracts from the Picual variety. It is worth noting that the in vitro studies employed heterogeneous osteoblast models, including immortalized or tumor-derived cell lines such as MG-63, which may overestimate anabolic responses due to their high proliferative capacity and altered gene expression profiles. Although useful for mechanistic screening, these models differ substantially from primary osteoblasts in metabolic behavior and responsiveness to stimuli. Therefore, findings obtained in such models should be interpreted with caution and validated in more physiologically relevant systems. Liu et al. [20] reported that oleuropein increased BMD and modulated the OPG/RANKL system, reducing inflammatory and oxidative markers in ovariectomized rats. Filip et al. [20,25] showed that 12-month supplementation with olive polyphenols stabilized BMD, increased osteocalcin levels in osteopenic women, and improved lipid profile.

### 4.3. Effects on the Skeletal Muscle Tissue

Four studies explored muscle-related effects. González-Hedström et al. [22] demonstrated that OLE supplementation in aged rats reduced sarcopenia-related biomarkers, including HDAC-4 and IL-6, while increasing myogenin, an important factor associated to skeletal muscle proliferation and differentiation. De Stefanis et al. [22,32] found that oleocanthal preserved myotube morphology and reduced the expression of atrophy-related genes (atrogin-1, MuRF1) in C2C12 cells under catabolic stress. Nardi et al. [33] revealed that oleuropein prevented oxidative stress-induced muscle cell death and restored MyoD expression. In a recent randomized controlled trial, Pinckaers et al. [24] reported that oleuropein supplementation (100 mg/day for 36 days) increased resting skeletal muscle pyruvate dehydrogenase (PDH) activity in older males, suggesting a potential effect on energy metabolism. However, the intervention did not lead to improvements in mitochondrial respiration, muscle strength, fatigue resistance, or body composition compared to placebo. These null results underscore the limited functional translation of the observed biochemical change and highlight the need for longer trials or combined lifestyle interventions to assess clinically relevant outcomes.

**Figure 4:**
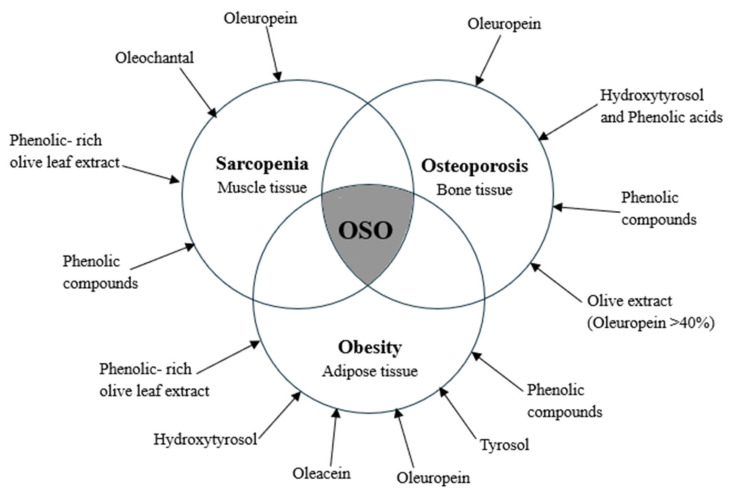
Overview of types of polyphenols that showed an effect on different tissues.

These findings underscore the tissue-specific biological activities of olive oil polyphenols and support their potential role as nutraceutical agents in preventing or managing osteosarcopenic obesity. Figure 4 provides a graphical overview of the main polyphenolic compounds and the specific tissue types—adipose, bone, and muscle—on which they exert their effects.

### 4.4. Risk of Bias Assessment for In Vivo Studies

The risk of bias in the included in vivo animal studies was assessed using SYRCLE’s Risk of Bias Tool, which evaluates ten domains adapted from the Cochrane Collaboration tool. These domains include selection bias (sequence generation, allocation concealment, baseline characteristics), performance bias (random housing, blinding of caregivers), detection bias (random outcome assessment, blinding of outcome assessor), attrition bias, reporting bias, and other sources of bias.

A color-coded heatmap (Figure 1) was generated to visualize the risk levels across studies and domains. Most studies showed a low risk of bias in domains related to sequence generation and baseline comparability. However, several domains—particularly allocation concealment, blinding of caregivers, and outcome assessors—were frequently rated as unclear, due to insufficient methodological details. One study presented a high risk of bias in random housing and caregiver blinding.

These results highlight the need for more transparent reporting and methodological rigor in preclinical animal studies evaluating olive oil polyphenols.

### 4.5. Risk of Bias Assessment for in Humans Studies

Among the three included randomized clinical trials, two were judged to have a low risk of bias across all domains. The remaining study [23] raised some concerns regarding the randomization process, although other domains were rated as low risk. In contrast, the trial by Filip et al. [25] showed some concerns in multiple domains, particularly in deviations from intended interventions and outcome reporting. A heatmap visualization of the RoB 2.0 assessment highlights these patterns across domains and studies (Figure 2).

### 4.6. Risk of Bias Assessment for In Vitro Studies

Out of the eight in vitro studies included, one study was judged to have a low risk of bias, five showed a moderate risk, and two—Stefanon and Colitti [27] and Garcia-Martínez et al. [31] —were considered at high risk of bias. In both cases, key methodological flaws included a lack of transparency in reporting replicates, absence of randomization or blinding, and insufficient detail regarding experimental controls. These limitations undermine reproducibility and increase the likelihood of bias in outcome assessment. All studies clearly described the cell models used and included appropriate control conditions. Most studies reported the number of replicates and employed valid statistical methods. However, none of the studies implemented randomization or blinding, and two studies lacked sufficient detail on replicates and protocol transparency, which led to their classification as high RoB [27,31].

To ensure a more robust and context-sensitive evaluation of in vitro methodological quality, we adopted an integrative approach combining domains from the ToxRTool (Toxicological data Reliability Assessment Tool) and the OHAT Risk of Bias Tool developed by the U.S. National Toxicology Program. The final assessment grid included ten domains spanning reporting quality (e.g., transparency in methods, validated protocols, and statistical appropriateness) and internal validity (e.g., use of randomization, blinding, and completeness of reporting). This hybrid method allowed us to better capture potential sources of bias in efficacy-related mechanistic studies. Similar adaptations have been recommended for evidence integration in environmental and nutritional health sciences [35]. Although originally developed for toxicological research, the ToxRTool and the OHAT Risk of Bias Tool have been increasingly applied to efficacy studies involving bioactive compounds. Their domains—such as reproducibility, appropriate controls, and methodological transparency—are essential for evaluating internal validity in both toxicological and therapeutic experimental settings. This approach has been used in prior reviews examining bioactivity of nutraceuticals in cellular models.

## 5. Discussion

This systematic review highlights the potential of olive oil polyphenols, such as hydroxytyrosol, oleuropein, oleocanthal, and oleacein, as modulators of pathophysiological processes in osteosarcopenic obesity (OSO). Across 15 studies (8 in vitro, 4 in vivo, 3 human), these compounds showed antioxidant, anti-inflammatory, anti-adipogenic, and osteo-/myo-protective effects on adipose, bone, and muscle tissues. Adipose tissue benefits included reduced inflammation and lipid accumulation via pathways like NF-κB (a protein complex regulating inflammation) and AMPK. Bone studies showed enhanced osteoblast proliferation and BMD stabilization. Muscle effects included reduced atrophy and improved mitochondrial activity [28,29].

These effects were further supported by in vivo studies, where hydroxytyrosol was associated with reductions in fat accumulation and systemic inflammation, consistent with in vitro evidence on adipocyte modulation, although not all endpoints were directly comparable [21,34]. Daily hydroxytyrosol supplementation in humans was associated with a reduction in android fat mass and improvements in lipid metabolism, as reported in a randomized controlled trial by Fytili et al. [23].

Bone-related studies demonstrated that olive oil phenolics enhance osteoblast proliferation and differentiation, upregulate osteogenic genes, and modulate remodeling pathways like OPG/RANKL [20,30]. Although clinical evidence points in a similar direction, it remains limited and preliminary. For instance, Filip et al. [25] reported stabilized bone mineral density and improved biochemical markers following long-term supplementation, but the small sample size (*n* = 64) limits the robustness and generalizability of these findings. Likewise, the study by Fytili et al. [23] included only 29 participants, further underscoring the need for larger, well-powered trials.

Regarding muscle tissue, both in vitro and in vivo findings revealed the ability of olive polyphenols to preserve myotube morphology, suppress proteolysis-related gene expression (atrogin-1, MuRF1), and restore mitochondrial activity and redox balance [32,33]. While human evidence is still limited, Pinckaers et al. [24] reported that oleuropein supplementation increased resting skeletal muscle PDH fractional activation from 0.19 ± 0.09 to 0.27 ± 0.09, compared to a stable value in the placebo group (0.19 ± 0.07), suggesting a modest but measurable enhancement in mitochondrial enzyme activity.

Compared to olive oil polyphenols, other food-derived compounds have been investigated for their potential to counteract the multifactorial decline observed in OSO. For example, omega-3 fatty acids (EPA and DHA), administered at 2 g/day or more for 12–24 weeks, have shown anabolic and anti-inflammatory effects on muscle and bone, particularly when combined with physical exercise. Vitamin D supplementation, typically 800 to 2000 IU/day, is associated with improved calcium metabolism and muscle function, although its effects on fat distribution and lean mass remain inconclusive. Collagen peptides, often used at 10 g/day for at least 12 weeks, have demonstrated benefits in increasing muscle mass and bone density, especially in elderly subjects engaged in resistance training. Isoflavones, naturally present in soy and administered at 40–80 mg/day, have been shown to improve bone health and reduce fat accumulation, particularly in postmenopausal women.

In this context, olive oil polyphenols such as hydroxytyrosol and oleuropein emerge as promising agents due to their combined antioxidant, anti-inflammatory, and tissue-protective effects. However, the clinical evidence supporting their efficacy is still limited, with existing trials using doses of approximately 15 mg/day for hydroxytyrosol or up to 250 mg/day of oleuropein-rich extracts, typically over 4 to 12 weeks. Further studies are needed to define optimal intake regimens and to assess long-term outcomes in at-risk populations.

Overall, the available literature supports the hypothesis that olive oil polyphenols may act as bioactive agents capable of intervening in the multifactorial etiology of OSO. However, the heterogeneous nature of experimental models, dosage regimens, and outcome measures underscores the need for standardized, large-scale clinical trials to validate efficacy and define optimal intervention strategies.

Notably, none of the included studies performed formal dose–response or duration–response analyses, representing a key limitation for clinical translation. The absence of such data hinders the identification of optimal intake thresholds and exposure periods for meaningful physiological effects in humans.

## 6. Limitations

Several limitations of this review should be acknowledged. First, most included studies were preclinical investigations, and while mechanistically informative, they may not translate directly to clinical outcomes in humans. The small number of human trials available—only three—limits the generalizability of findings. Second, the multiplicity in polyphenol types, dosages, intervention durations, and outcome measures introduces substantial heterogeneity of the results, complicating direct comparisons and meta-analytical synthesis. Third, the absence of standardized diagnostic criteria for OSO across studies reduces comparability. In addition, the literature search was restricted to PubMed and Google Scholar, which, while targeted and sufficient for the present scope, may have introduced selection bias. Future systematic reviews could benefit from including broader biomedical and interdisciplinary databases such as Embase, Scopus, or Cochrane CENTRAL to ensure more comprehensive coverage. Finally, due to the heterogeneity of study designs (in vitro, animal, and human) and the small number of clinical trials, formal assessment of publication bias (e.g., via funnel plots or Egger’s test) was not feasible. However, we cannot exclude the possibility of selective reporting or publication bias, especially considering the predominance of small, single-center studies with positive outcomes.

## 7. Practical Applications

These findings suggest that olive oil polyphenols may support OSO prevention, particularly in aging populations, if their effects are confirmed in larger, well-powered clinical trials. While extra virgin olive oil remains a staple of the Mediterranean diet with known health benefits, the use of isolated polyphenol supplements should be approached with caution until more robust evidence is available. Future research should prioritize adequately powered randomized controlled trials employing validated OSO diagnostic criteria to assess the clinical efficacy of olive oil polyphenols in relevant population.

## 8. Conclusions

Olive oil polyphenols, particularly hydroxytyrosol and oleuropein, show preliminary evidence of modulating surrogate biochemical markers related to bone and skeletal muscle metabolism results by targeting inflammation, oxidative stress, adipogenesis, osteoblast differentiation, and muscle atrophy. While preclinical evidence is relatively robust and coherent, limited human trials highlight the need for further research. Standardized RCTs with clear endpoints and OSO-specific frameworks, especially in at-risk populations (e.g., non-sarcopenic obese aged adults), are essential to translating these findings into evidence-based dietary recommendations, such as increased EVOO intake or polyphenol supplementation, for aging populations.

## Figures and Tables

**Table 1:** (**A**). Summary of studies using purified olive oil polyphenols on OSO-related outcomes. (**B**). Summary of studies using crude mixtures (olive leaf or EVOO extracts).

A						
**Author, Year**	**Model**	**Compound**	**Sample**	**Dose**	**Mechanism of Action**	**Key Findings**
Stefanon & Colitti, 2016 [27]	In vitro	Hydroxytyrosol	Human adipocytes	5–70 mg/mL	↓ Triglycerides, ↑ apoptosis	Anti-adipogenic, pro-lipolytic
Pacifici et al., 2020 [29]	In vitro	Tyrosol	3T3-L1 cells	300–500 μM	↓ Adipogenesis, ↑ lipolysis	AMPK-ATGL-HSL pathway activation
Carpi et al., 2019 [28]	In vitro	Oleocanthal, Oleacein	Human preadipocytes	25 μmol/L	↓ NF-κB activation, ↓ miRNA	Anti-inflammatory
De Stefanis et al., 2024 [32]	In vitro	Oleocanthal	C2C12 myotubes	10 μM	↓ Atrogin-1, MuRF1	Myotube preservation
Nardi et al., 2020 [33]	In vitro	Oleuropein (aglycone)	C2C12 myocytes	10 μM	↓ Oxidative stress	↑ MyoD, anti-atrophic
Liu et al., 2022 [20]	In vivo	Oleuropein	OVX rats	200 μg/kg	↑ BMD, ↓ IL-6/TNF-α	Bone remodeling via OPG/RANKL
Liu et al., 2019 [34]	In vivo	Hydroxytyrosol	Mice	50 mg/kg	Modulation of gut microbiota	Anti-obesogenic effect
Fytili et al., 2022 [23]	Human	Hydroxytyrosol	Overweight women	5–15 mg/day	↓ Adipogenesis gene expression	↓ Visceral fat
Pinckaers et al., 2025 [24]	Human	Oleuropein	Older males	100 mg/day	↑ PDH activity	Muscle metabolism support
**B**						
**Author, Year**	**Model**	**Extract Type**	**Sample**	**Dose**	**Mechanism of Action**	**Key Findings**
Lepore et al., 2019 [19]	In vitro/in vivo	Oleacein (from olive extract)	3T3-L1 cells, mice	10–100 µM; 20 mg/kg	↓ Lipid accumulation, ↑ adiponectin	↓ Fat mass, anti-inflammatory
Melguizo-Rodríguez et al., 2019 [30]	In vitro	EVOO phenolics	MG63 osteoblasts	10^−6^ M	↑ TGFβ1, BMP2, BMP7	Osteogenic effect
Garcia-Martínez et al., 2016 [31]	In vitro	EVOO phenolics	MG63 cells	Various	↑ ALP, ↑ proliferation	Variety-dependent effect
González-Hedström et al., 2021 [22]	In vivo	Olive Leaf Extract (OLE)	Aged rats	100 mg/kg	↓ IL-6, ↑ myogenin	Anti-sarcopenic, ↑ insulin sensitivity
Fki et al., 2020 [21]	In vivo	HT- and Oleuropein-rich extracts	Rats	16 mg/kg	↓ adipogenesis, ↑ lipid metabolism	Hepatoprotective effects
Filip et al., 2015 [25]	Human	Polyphenol extract (oleuropein >40%)	Osteopenic women	250 mg/day	↑ Osteocalcin, ↑ BMD	Improved bone/lipid profile

**↓**: decrease or downregulation; **↑**: increase or upregulation in levels.

## Data Availability

Any inquiry can be directed to the corresponding author.
